# Real-time polymerase chain reaction analysis of *MDM2* and *CDK4* expression using total RNA from core-needle biopsies is useful for diagnosing adipocytic tumors

**DOI:** 10.1186/1471-2407-14-468

**Published:** 2014-06-26

**Authors:** Taro Sasaki, Akira Ogose, Hiroyuki Kawashima, Tetsuo Hotta, Hiroshi Hatano, Takashi Ariizumi, Hajime Umezu, Riuko Ohashi, Tsuyoshi Tohyama, Naohito Tanabe, Naoto Endo

**Affiliations:** 1 Division of Orthopedic Surgery, Niigata University Graduate School of Medical and Dental Sciences, 757-1, Asahimachi-dori, Niigata City, Niigata 951-8510, Japan; 2 Division of Pathology, Niigata University Medical and Dental Hospital, Niigata, Japan; 3 Departments of Orthopedic Surgery, Niigata Cancer Center Hospital, Niigata, Japan; 4 Center of Molecular Biology and Cytogenetics, SRL, Inc, Tokyo, Japan; 5 Department of Health and Nutrition, Faculty of Human Life Studies, University of Niigata Prefecture, Niigata, Japan

**Keywords:** Liposarcoma, Atypical lipomatous tumor, Adipocytic tumors, MDM2, CDK4, Real-time PCR

## Abstract

**Background:**

Diagnosing adipocytic tumors can be challenging because it is often difficult to morphologically distinguish between benign, intermediate and malignant adipocytic tumors, and other sarcomas that are histologically similar. Recently, a number of tumor-specific chromosome translocations and associated fusion genes have been identified in adipocytic tumors and atypical lipomatous tumors/well-differentiated liposarcomas (ALT/WDL), which have a supernumerary ring and/or giant chromosome marker with amplified sequences of the *MDM2* and *CDK4* genes. The purpose of this study was to investigate whether quantitative real-time polymerase chain reaction (PCR) could be used to amplify *MDM2* and *CDK4* from total RNA samples obtained from core-needle biopsy sections for the diagnosis of ALT/WDL.

**Methods:**

A series of lipoma (n = 124) and ALT/WDL (n = 44) cases were analyzed for cytogenetic analysis and lipoma fusion genes, as well as for *MDM2* and *CDK4* expression by real-time PCR. Moreover, the expression of *MDM2* and *CDK4* in whole tissue sections was compared with that in core-needle biopsy sections of the same tumor in order to determine whether real-time PCR could be used to distinguish ALT/WDL from lipoma at the preoperative stage.

**Results:**

In whole tissue sections, the medians for *MDM2* and *CDK4* expression in ALT/WDL were higher than those in the lipomas (P < 0.05). Moreover, karyotype subdivisions with rings and/or giant chromosomes had higher *MDM2* and *CDK4* expression levels compared to karyotypes with 12q13-15 rearrangements, other abnormal karyotypes, and normal karyotypes (P < 0.05). On the other hand, *MDM2* and *CDK4* expression levels in core-needle biopsy sections were similar to those in whole-tissue sections (*MDM2*: P = 0.6, *CDK4*: P = 0.8, Wilcoxon signed-rank test).

**Conclusion:**

Quantitative real-time PCR of total RNA can be used to evaluate the *MDM2* and *CDK4* expression levels in core-needle biopsies and may be useful for distinguishing ALT/WDL from adipocytic tumors. Thus, total RNA from core-needle biopsy sections may have potential as a routine diagnostic tool for other tumors where gene overexpression is a feature of the tumor.

## Background

Adipocytic tumors represent the largest group of soft tissue tumors [1]. The diagnosis of adipocytic tumors is primarily based on clinical features and histologic patterns [2]. However, the distinction between lipomas and atypical lipomatous tumors/well-differentiated liposarcomas (ALT/WDL) may be difficult to distinguish morphologically.

Cytogenetic studies of adipocytic tumors have revealed a clear association between chromosomal findings and clinicohistopathological features [3,4]. Clonal chromosome aberrations have been found in nearly 60% of all lipomas [4], of which two-thirds are rearrangements involving the 12q13-15 chromosomal region. A variety of rearrangements, mainly involving the 6p and 13q regions, are observed in the remaining lipoma cases [5-7]. In tumors with aberrations involving 12q13-15 region, the high mobility group protein gene (*HMGA2*, also known as *HMGIC*) on chromosome 12 is rearranged. These aberrations may also result in the creation of chimeric genes, in which the *HMGA2* gene is fused to multiple genes. The most frequent gene aberration in lipomas is *HMGA2/LPP *[8].

ALT/WDL and dedifferentiated liposarcomas (DDL) most often have a supernumerary ring and giant marker chromosomes composed of amplified sequences from the 12q13-15 region [9,10], including the murine double-minute type 2 gene (*MDM2*) and the cyclin-dependent kinase 4 gene (*CDK4*) [11-13]. Amplification of the 12q13-15 region has not been observed in lipoma, and the MDM2 and CDK4 proteins are known to be overexpressed in ALT/WDL but not in lipoma [14]. Immunohistochemistry for MDM2 and CDK4 plays a helpful role in the differential diagnosis of adipocytic tumors. Aleixo et al. [15] reported that MDM2 has very high sensitivity (100%) in the identification of ALT/WDL among lipomas, but has low specificity (58.8%), whereas CDK4 has low sensitivity (68.4%), but high specificity (88.2%). Immunohistochemistry may be used to demonstrate *MDM2* and *CDK4* amplification, but the sections sometimes show several staining patterns such as diffuse, moderate, and focal positivity. Categorization of these staining patterns has been developed differently by different researchers, making it difficult to compare studies effectively.

The use of minimally invasive biopsies to diagnose soft tissue tumors has become increasingly common. On the other hand, ALT/WDL can be difficult to distinguish morphologically from benign lipomatous lesions, especially with limited material in which the diagnostic features of scattered atypical cells are not present because of heterogeneity of the neoplasm. However, distinguishing benign lipomatous tumors from ALT/WDL is important at primary biopsy.

In this study, we used whole tissue sections from surgically resected specimens to retrospectively analyze cytogenetic findings by quantifying *MDM2* and *CDK4* expression levels in lipomas and ALT/WDL with real-time polymerase chain reaction (PCR) from total RNA. We evaluated the clinical utility of measuring *MDM2* and *CDK4* expression levels to establish a diagnosis of adipocytic tumors, with the aim of making a distinction between lipoma and ALT/WDL. Moreover, we compared the results of *MDM2* and *CDK4* expression in whole tissue sections with those in core-needle biopsy sections in order to investigate whether real-time PCR for *MDM2* and *CDK4* could be used to distinguish between ALT/WDL and lipoma prior to surgery.

## Methods

### Specimens

Tumor samples were obtained from patients that underwent surgical resection at Niigata University Hospital between August 2001 and December 2012. In total, 124 cases of lipoma and 44 cases of ALT/WDL were studied (Additional file 1: Table S1). In all cases, the diagnosis of lipoma or ALT/WDL was established according to the World Health Organization (WHO) Classification of Tumors [2] by using hematoxylin and eosin-stained tissue sections from the surgical resection specimens. Two experienced pathologists independently reviewed the cases in which it was difficult to distinguish between lipoma and ALT/WDL. There were 159 primary and 9 recurrent tumors. The patient cohort consisted of 96 men and 72 women between 24 and 86 years of age (mean 59.0 years; range 24–86 years).

The samples were taken from both core-needle biopsy sections and whole tissue sections of the adipose tissue tumors. Some of the samples represent paired whole tissue sections and core-needle biopsy sections from the same tumor. Core-needle biopsy sections were sampled prior to or after surgical resection using a 16G Tru-Cut trocar with at least two passes or until an adequate sample was obtained.

### Cytogenetic analysis

The tumor specimens that were analyzed were obtained immediately after surgical excision. Portions of the tumor were treated with collagenase and cultured at 37°C for 4 days. The chromosome slides were prepared from short-term-cultured tumor cells using the standard trypsin Giemsa banding technique. Karyotypes were described on the basis of the short system of the International System for Human Cytogenetic Nomenclature (ISCN) [16]. The karyotypes were classified as either normal or abnormal. The abnormal karyotypes were further subdivided according to the presence of a rearrangement in 12q13-15, rearrangement or loss of chromosome 13q, rearrangement of 6p21-23, and the presence of a supernumerary ring and/or giant marker chromosome, as well as other aberrations [4-6]. Some tumors had more than one of these aberrations and were thus included in more than one subgroup.

### Reverse transcription PCR

Total RNA was prepared using Isogen reagent (Nippon Gene; Tokyo, Japan) from core-needle biopsy sections according to the manufacturer’s recommendations. Synthesis of cDNA was performed using a PrimeScript™ RT reagent kit (TaKaRa Bio; Tokyo, Japan), and PCR was performed with rTaq DNA Polymerase (Toyobo; Osaka, Japan). Glyceraldehyde 3-phosphate dehydrogenase (GAPDH: forward; 5′TGAAGGTCGGAGTCAACGGATTTGGT 3′, reverse; 5′CATGTGGGCCATGAGGTCCACCAC 3′) was used as the internal control for uniform RNA loading. The primers that were used to detect *HMGA2* transcripts are listed in Additional file 1: Table S2 as *HMGA2/LPP*, *HMGA2/RDC1*, and *HMGA2/NFIB *[17]. The PCR conditions used were as follows: the reaction mixture was heated for 3 min at 94°C, followed by 30 cycles of 30 s denaturation at 94°C, 30 s annealing at 55 °C, and a 30 s extension at 72°C using a PTC-200 Peltier Thermal Cycler (MJ Research; Waltham, MA, USA). PCR products were analyzed by electrophoresis on a 1.5% agarose gel containing ethidium bromide, and were photographed under ultraviolet light.

### Quantitative real-time PCR

RNA samples were taken from both core-needle biopsy sections and whole-tissue sections. Total RNA and synthesis of cDNA were prepared as described above. Quantitative real-time PCR was performed using SYBR Premix Ex Taq II in a Thermal Cycler Dice Real Time System TP800 (TaKaRa Bio; Otsu, Japan). The primers of target genes used for this analysis were MDM2 and CDK4, and the primer sequences are listed in Additional file 1: Table S3. *GAPDH* was selected as the reference gene (forward; 5′ GCACCGTCAAGGCTGAGAAC 3′, reverse; 5′ TGGTGAAGACGCCAGTGGA3′). The gene copy numbers of *MDM2* and *CDK4* were calculated by using a standard curve that was constructed using the NDDLS-1 cell line [18]. The level of expression for the target gene was calculated as the ratio of the copy number of the target gene (*MDM2* or *CDK4*) to that of the reference gene (*GAPDH*). Total RNA from normal human adipose tissue was purchased from BioChain (Newark, CA, USA), and used as a calibrator. Finally, the relative level of expression was calculated as follows: [copy number of the target gene (*MDM2* or *CDK4*)/copy number of the reference gene (*GAPDH*)]/copy number of the target gene (*MDM2* or *CDK4*) in normal adipose tissue.

### Statistical analysis

Results from quantitative real-time PCR are reported as the median of *MDM2* and *CDK4* relative expression levels. The Mann–Whitney U test was used to compare differences in *MDM2* and *CDK4* median relative expression levels between lipoma and ALT/WDL. The Steel-Dwass test was used for comparison of differences in each of the subdividing karyotypes. *MDM2* and *CDK4* relative expression levels in the core-needle biopsy sections were compared to those in the whole-tissue sections by the Wilcoxon signed-rank test and Spearman rank correlation coefficient. P values < 0.05 were considered to be statistically significant.

### Consent

The study complies with the Declaration of Helsinki and was approved by the Institutional Review Board of Niigata University Hospital. Written informed consent was obtained from each patient before the specimens were taken in accordance with the local ethics committee (Niigata University Hospital).

## Results

### Cytogenetic findings

Cytogenetic analysis was performed on 104/168 cases (66 lipoma cases and 38 ALT/WDL cases). Table 1 shows the results from the clinical and cytogenetic analyses of the lipomas, which indicate that an abnormal karyotype was present in 56 of the lipoma cases (85%). By subdividing the karyotypes into previously identified cytogenetic subgroups, it was discovered that 21 lipomas had a 12q13-15 rearrangement (38%), 6 had a 13q rearrangement or loss of chromosome 13 (11%), 3 had a 6p21-23 rearrangement (5%), 4 had one or more ring chromosomes (7%), and 25 had other rearrangements (45%). In addition, 10 cases of lipoma (15%) had a normal karyotype.

**Table 1:** Clinical and cytogenetic findings in lipomas

	**Sex**		**Age (years)**			**Location**				**Total**
**Karyotype**	**M**	**F**	**20-40**	**40-60**	**>60**	**U**	**L**	**T**	**H**	**= 66**
**Normal**	6	4	2	6	2	1	1	3	5	10 (15%)
**Abnormal**	36	20	5	23	28	13	17	17	9	56 (85%)
*Ring/Giant chromosome*	*1*	*3*	*0*	*1*	*3*	*1*	*2*	*1*	*0*	*4 (7%)*
*12q13-15 rearrangement*	*13*	*8*	*0*	*8*	*13*	*6*	*7*	*7*	*1*	*21 (38%)*
*13q rearrangement*	*5*	*1*	*0*	*2*	*4*	*0*	*1*	*1*	*4*	*6 (11%)*
*6p21-23 rearrangement*	*2*	*1*	*1*	*0*	*2*	*1*	*0*	*1*	*1*	*3 (5%)*
*Other*	*17*	*8*	*4*	*13*	*8*	*5*	*8*	*7*	*5*	*25 (45%)*

*Abbreviations:M* male, *F* female, *U* upper extremity, *L* lower extremity, *T* trunk, *H* head and neck. Note that some cases showed more than one karyotypic aberration.

Analysis of ALT/WDL (Table 2) demonstrated that 36 ALT/WDL (95%) cases had an abnormal karyotype while the remaining 2 cases (5%) had a normal karyotype. Subdividing the karyotypes showed that most of the abnormal karyotypes had ring and/or giant chromosomes; 15 ALT/WDLs had one or more rings and/or giant chromosomes (42%), 5 had a 12q13-15 rearrangement (14%), 5 had a 13q rearrangement or loss of chromosome 13 (14%), 3 had a 6p21-23 rearrangement (8%), and 10 had other rearrangements (28%).

**Table 2:** Clinical and cytogenetic findings in atypical lipomatous tumors/well-differentiated liposarcomas

	**Sex**		**Age (years)**			**Location**				**Total**
**Karyotype**	**M**	**F**	**20-40**	**40-60**	**>60**	**U**	**L**	**T**	**H**	**= 38**
**Normal**	1	1	0	1	1	0	2	0	0	2 (5%)
**Abnormal**	21	15	2	16	18	6	20	8	2	36 (95%)
*Ring/Giant chromosome*	*7*	*8*	*1*	*5*	*9*	*1*	*9*	*5*	*0*	*15 (42%)*
*12q13-15 rearrangement*	*4*	*1*	*0*	*2*	*3*	*1*	*3*	*0*	*1*	*5 (14%)*
*13q rearrangement*	*4*	*1*	*0*	*3*	*2*	*3*	*1*	*1*	*0*	*5 (14%)*
*6p21-23 rearrangement*	*1*	*2*	*0*	*2*	*1*	*1*	*1*	*0*	*1*	*3 (8%)*
*Other*	*5*	*5*	*1*	*3*	*6*	*1*	*7*	*2*	*0*	*10 (28%)*

*Abbreviations:M* male, *F* female, *U* upper extremity, *L* lower extremity, *T* trunk, *H* head and neck. Note that some cases showed more than one karyotypic aberration.

### *HMGA2* fusion genes

Reverse transcription PCR was used to evaluate 128/168 samples (96 lipoma samples and 32 ALT/WDL samples) (Table 3). The *HMGA2/LPP* gene fusion transcript was detected in 10 samples (8%) while the *HMGA2/RDC1* fusion transcript was only detected in 3 samples (2%). No sample expressed the *HMGA2/NFIB* fusion gene. Most of these cases were categorized as lipomas, except for one *HMGA2/LPP* case, which was diagnosed as ALT/WDL. Cytogenetic analysis of the 6 cases that tested positive for *HMGA2/LPP* revealed that 5 of them had a t(3;12)(q27-28;q13-15) translocation that fused the *HMGA2* and *LPP* genes.

**Table 3:** **Reverse transcription PCR of *HMGA2 *fusion genes**

	**Lipoma**	**ALT/WDL**
*HMGA2-LPP*(+)	9 (9%)	1 (3%)
*HMGA2-RDC1*(+)	3 (3%)	0 (0%)
*HMGA2-NFIB*(+)	0 (0%)	0 (0%)
Fusion gene(-)	85 (88%)	31 (97%)

### *MDM2* and *CDK4* expression in whole tissue sections

**Figure 1:**
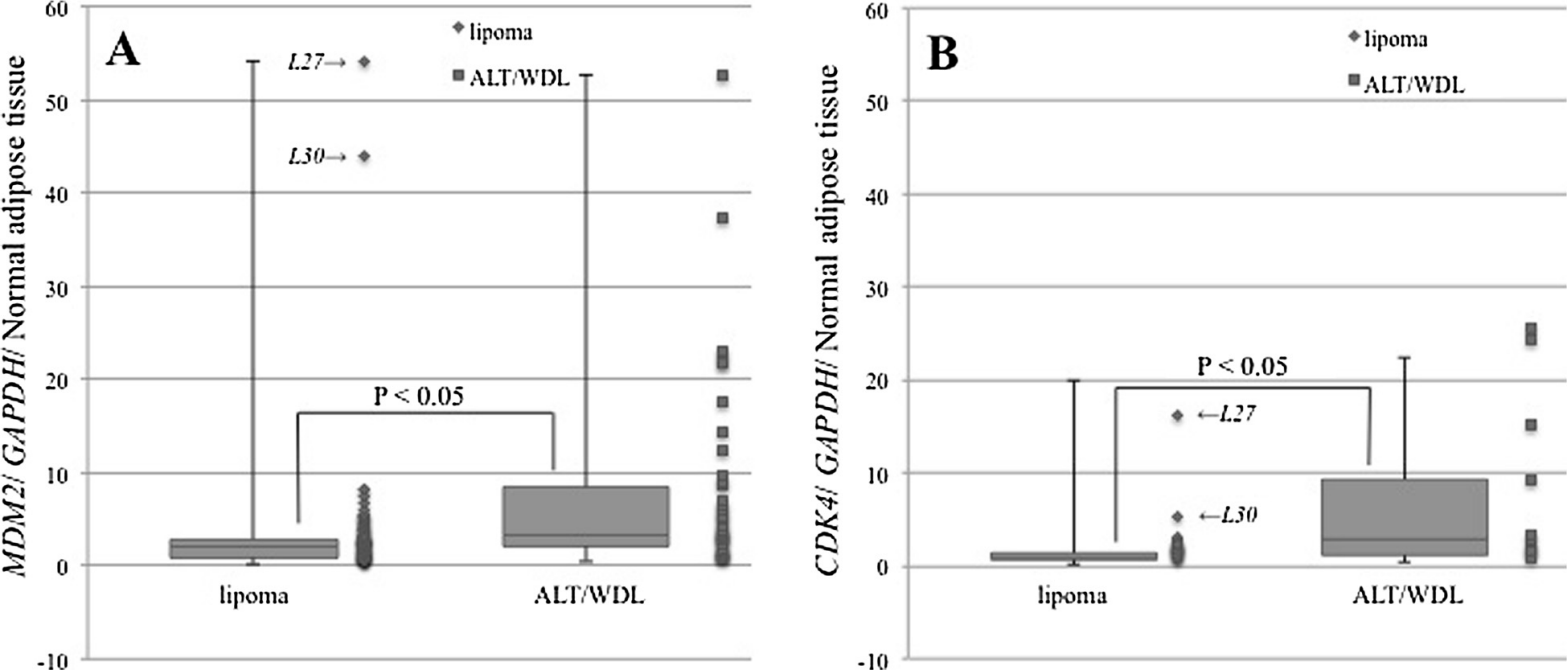
**Amplification of target genes from whole tissue sections by real-time PCR (A: *MDM2*, B: *CDK4*). ***Abbreviations:* L, lipoma; ALT/WDL, atypical lipomatous tumors/well-differentiated liposarcomas.

The gene expression levels of *MDM2* and *CDK4* were studied in 149/168 whole tissue sections (108 lipoma samples and 41 samples from the 38 cases of ALT/WDL). The medians for *MDM2* relative expression levels were 2.0 (range, 0.2–54.1) for lipoma and 3.4 (range, 0.4–52.5) for ALT/WDL. The medians for *CDK4* relative expression levels were 1.0 (range, 0.1–19.9) for lipoma and 2.9 (range, 0.4–22.4) for ALT/WDL (Figure 1). Both *MDM2* and *CDK4* relative expression levels in ALT/WDL were higher than those in lipoma (P < 0.05, Mann–Whitney U test).

**Figure 2:**
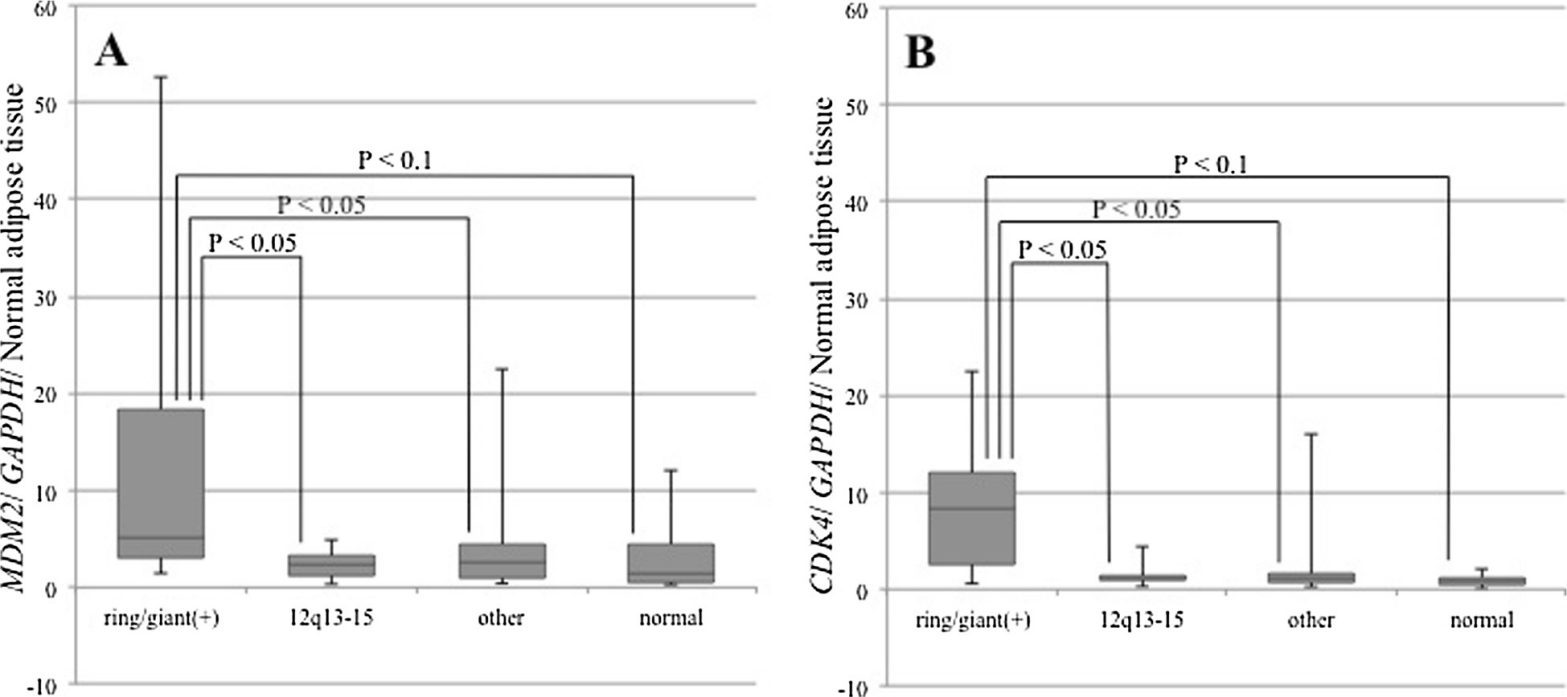
***MDM2 *** **and *CDK4 *** **amplification in subdividing karyotypes of whole tissue sections (A: *MDM2*, B: *CDK4*).**

In each of the subdividing karyotypes, the medians for relative *MDM2* expression were 5.1 (range, 3.1–52.5) for the 16 samples with a ring and/or giant chromosomes (3 lipoma samples and 13 ALT/WDL samples), 2.3 (range, 1.0–5.0) for the 23 samples with 12q13-15 rearrangements (19 lipoma samples and 4 ALT/WDL samples), 2.6 (range, 0.4–22.4) for the 34 samples with other rearrangements (21 lipoma samples and 13 ALT/WDL samples), and 1.5 (range, 0.2–12.0) for the 9 samples with a normal karyotype. The medians for *CDK4* expression were 8.4 (range, 0.9–22.4) for the 16 samples with ring and/or giant chromosomes, 1.1 (range, 0.3–4.5) for the 23 samples with 12q13-15 rearrangements, 1.1 (range, 0.2–16.0) for the 34 samples with other rearrangements, and 1.0 (range, 0.1– 2.1) for the 9 samples with a normal karyotype (Figure 2). Relative *MDM2* and *CDK4* expression levels in lipoma and ALT/WDL cases with a ring and/or giant chromosome were higher than those with 12q13-15 rearrangements and other abnormal karyotypes (P < 0.05, Steel-Dwass test). However, expression levels of cases with a ring and/or giant chromosome were not significantly higher than those with normal karyotypes (P < 0.1, Steel-Dwass test), because of the small number of samples with normal karyotypes.

### *MDM2* and *CDK4* expression in core-needle biopsy sections

**Figure 3:**
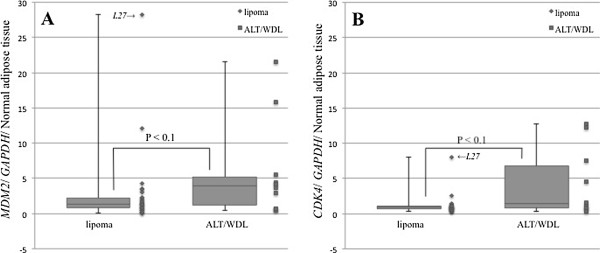
**Amplification of target genes from core-needle biopsy sections by real-time PCR (A: *MDM2*, B: *CDK4*). ***Abbreviations:* L, lipoma; ALT/WDL, atypical lipomatous tumors/well-differentiated liposarcomas.

**Figure 4:**
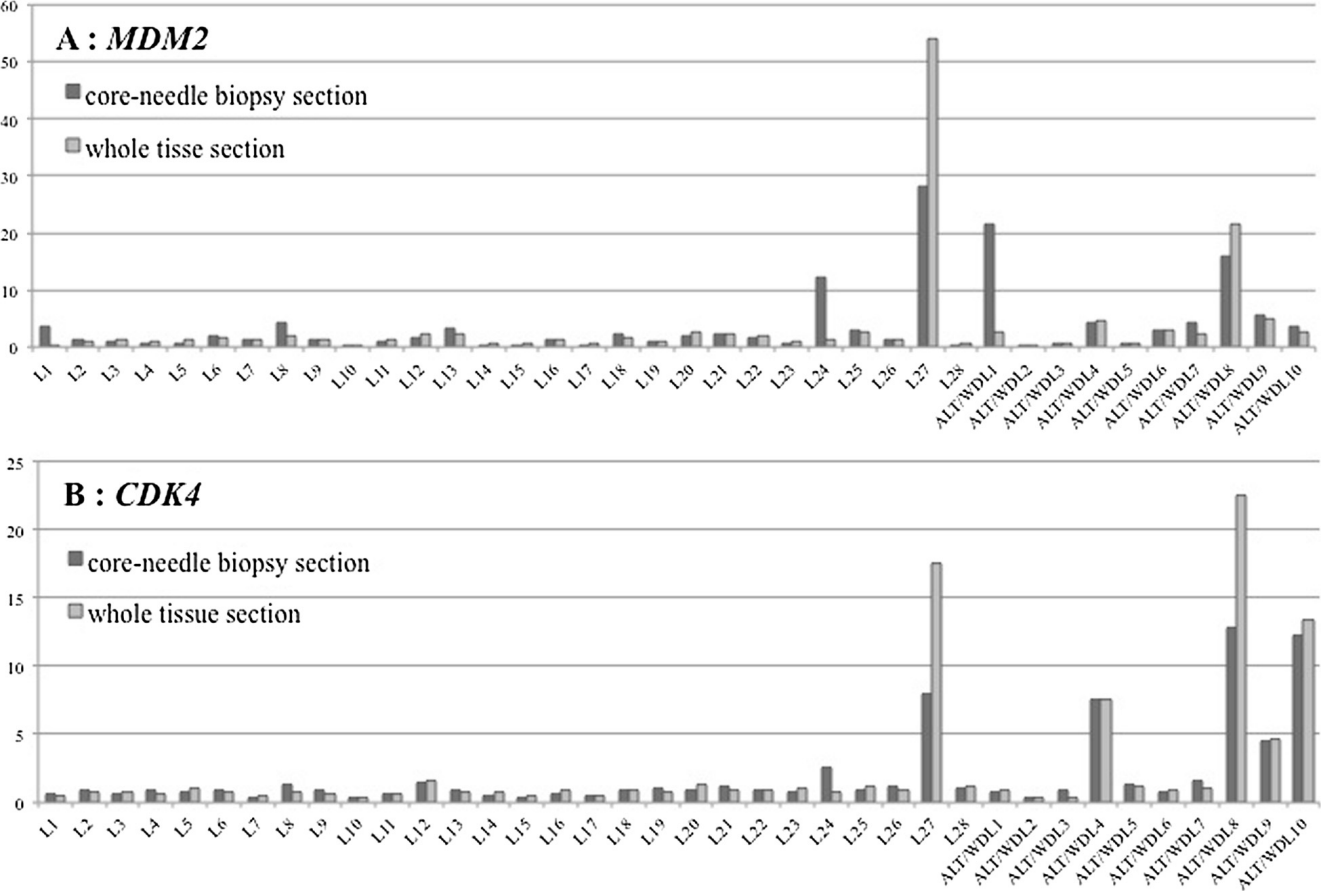
**Comparison of real-time PCR results from core-needle biopsy sections and whole tissue sections (A: MDM2, B: CDK4). ***Abbreviations: L* lipoma, *ALT/WDL* atypical lipomatous tumors/well-differentiated liposarcomas.

The relative gene expression levels of *MDM2* and *CDK4* were studied in 38/168 samples (28 lipoma samples and 10 ALT/WDL samples) from core-needle biopsy sections. The medians for relative *MDM2* expression were 1.3 (range, 0.1–28.2) for lipoma and 3.9 (range, 0.4–21.6) for ALT/WDL. The medians for relative *CDK4* expression were 0.9 (range, 0.3–8.0) for lipoma and 1.4 (range, 0.3–12.8) for ALT/WDL (Figure 3). Both *MDM2* and *CDK4* expression levels in core-needle biopsy sections showed no significant difference between lipoma and ALT/WDL (*MDM2*: P < 0.1, *CDK4*: P < 0.1, Mann–Whitney U test). *MDM2* and *CDK4* expression levels in the core-needle biopsy sections were comparable to those in the whole-tissue sections (*MDM2*: P = 0.6, *CDK4*: P = 0.8, Wilcoxon signed-rank test) (*MDM2*: ρ = 0.827, P = 0.000001, *CDK4*: ρ = 0.746, P = 0.000001, Spearman rank correlation coefficient) (Figure 4).

## Discussion

In the WHO classification, ALT/WDL is considered an intermediate (locally aggressive) malignancy. It accounts for approximately 40–45% of all liposarcomas and mostly occurs in the deep soft tissue of the extremities, especially in the thigh, retroperitoneum, and paratesticular areas. ALT/WDL mostly occurs in middle-aged and older individuals. Histologically, the tumor is composed either entirely or partially of mature adipocytic proliferation showing significant variation in cell size and, at least focal, nuclear atypia in both adipocytes and stromal cells. In some situations, ALT/WDL may be indistinguishable from benign adipocytic tumors at the histological level, and evaluation of inadequate samples can lead to misdiagnosis.

Lipomatous tumors are cytogenetically heterogeneous. Of the more than 200 cases with karyotypic abnormalities that have been described to date, most cytogenetic aberrations have been found to correlate with morphological subtype. In the present study, 36 out of the 38 (95%) ALT/WDL cases had an abnormal karyotype, whereby the ring and/or giant marker chromosome was identified in 15 of them (42 %). Fletcher et al. [3] reported that 29 of 37 (78 %) ALT cases (including 5 dedifferentiated cases) had a ring chromosome. In ordinary lipoma, however, the presence of a supernumerary ring chromosome is a rare finding [3,7,11]. It is interesting that tumors diagnosed as ordinary lipomas occasionally display rings and/giant chromosomes, which were found in 3% [3], 6% [5], and 2% [6] of ordinary lipoma samples in three different studies. The patients with ring chromosomes often have deep-seated lipomas that are, on average, larger and older than the other lipomas [1,5]. Furthermore, Bartuma et al. reported that it is interesting that in the 5 local recurrences among the 272 cases, 2 of the 5 cases that contained ring chromosomes were recurrent compared to 3/257 lipomas without ring chromosomes [5].

Ordinary lipoma is the most common soft tissue tumor and may appear at any site. It occurs mainly between 40 and 60 years of age and is more frequent in obese individuals [1]. Ordinary lipomas usually present as painless, slowly growing soft tissue masses, and can arise within subcutaneous tissue or within deep soft tissue or even on the surfaces of bone. The 12q13-15 region is the most common gene alteration involved in such aberrations, followed by the 6p21-23 and 13q rearrangements [5,6,8,19]. This chromosomal region has been found to recombine with a large number of bands through translocations. The most frequent translocation is t(3;12)(q27-28;q13-15), which fuses the *HMGA2* and *LPP* genes. This particular translocation is seen in more than 20% of tumors with 12q13-15 aberrations.

**Figure 5:**
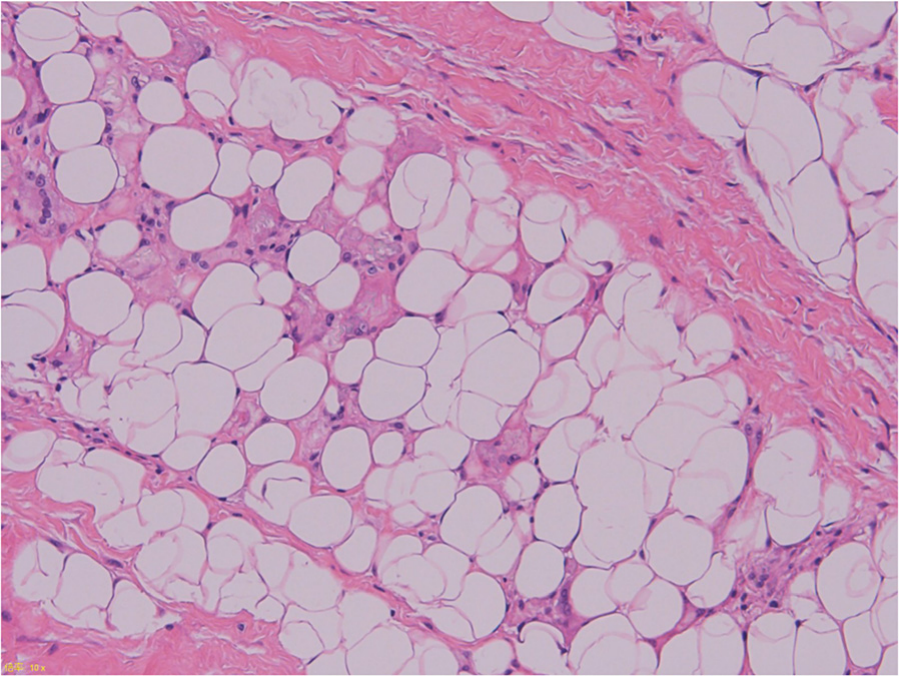
Variation in adipocytic size and extensive collagenous stroma were observed.

In this study, an abnormal karyotype was found in many more cases (85%), and rearrangements in the 12q13-15 region were found in lower frequency than previously described. In addition, the *HMGA2/LPP* gene fusion transcript was detected by reverse transcription PCR in 10 samples (8%). Hatano et al. [17] reported that the *HMGA2/LPP* gene fusion transcript was present in 23 of 102 cases (22.5%). Some of the discrepancies between our results and theirs may be due to the fact that there was a higher proportion of older patients in our study. There was one case of *HMGA2/LPP* diagnosed as ALT/WDL, which was a deep-seated adipocytic tumor in the ankle. Histopathologically, there were variations in adipocytic cell size and extensive septa, but upon further review, few hyperchromatic stromal cells were observed (Figure 5). Furthermore, this case had a 12q13-15 rearrangement, which was confirmed by cytogenetic analysis, and *MDM2* and *CDK4* amplification was not detected by quantitative real-time PCR. It is possible that this case was a lipoma cytogenetically.

ALT/WDL is characterized by the presence of a supernumerary ring and/or a giant marker chromosome that contains an amplification of the 12q13-15 region, including the *MDM2* and *CDK4* genes [11-13,20]. This 12q13-15 amplification is not observed in benign adipocytic tumors, and therefore, its detection can be used as an ancillary diagnostic technique for the diagnosis of ALT/WDL [21,22]. Fluorescence *in situ* hybridization (FISH) analysis is a potential tool for showing *MDM2* and *CDK4* gene amplification. Weaver et al. [23] demonstrated that detection of *MDM2* amplification by FISH is a more sensitive and specific adjunctive test compared to MDM2 immunohistochemistry when aiming to differentiate ALT/WDL from various benign lipomatous tumors, especially if there are limited tissue samples.

**Figure 6:**
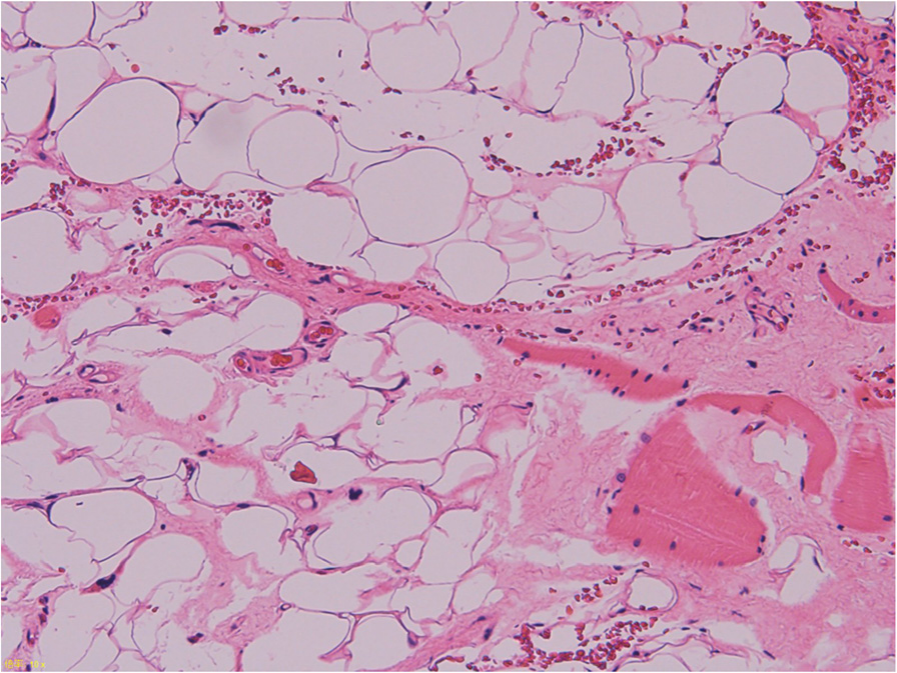
Sample L27 showed an infiltrative pattern with mature adipocytes and a few hyperchromatic stromal cells within fibrous septa.

**Figure 7:**
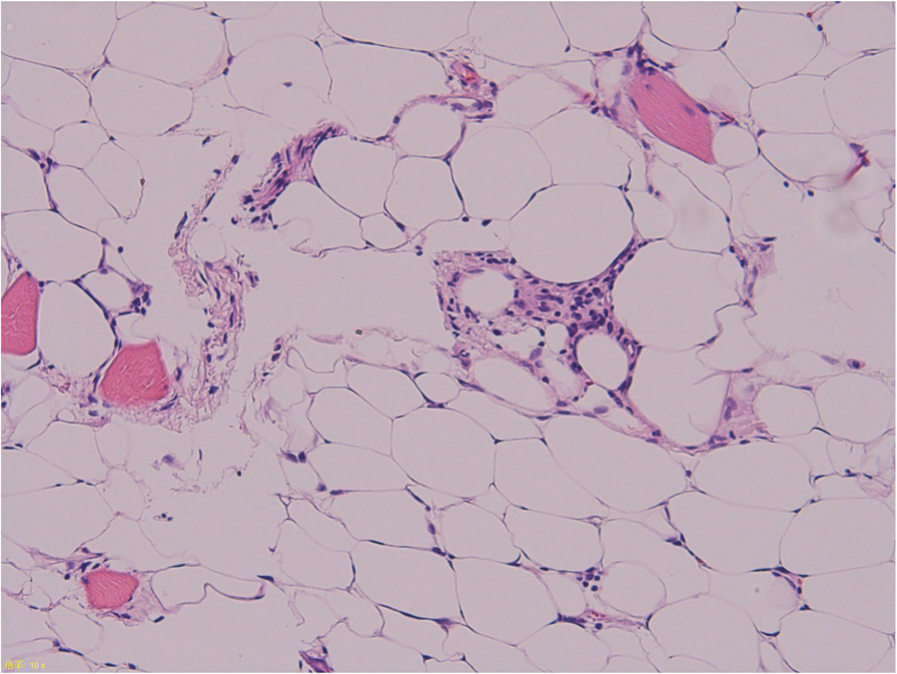
Sample L30 was a superficial intramuscular lipoma composed of mature adipocytes.

In this study, *MDM2* and *CDK4* expression levels, as determined by real-time PCR, were higher in ALT/WDL than in lipoma samples in whole tissue sections (P < 0.05) (Figure 1). Moreover, the expression levels from adipocytic tumors with rings and/or giant marker chromosomes were significantly higher compared to those from other aberrations (P < 0.05) (Figure 2). However, there were some lipomas with *MDM2* and *CDK4* amplification, cases L27 (*MDM2* 54.1, *CDK4* 17.5) and L30 (*MDM2* 43.8, *CDK4* 19.9), as shown in Figure 1. L27 was a deep-seated intramuscular lipoma in the thigh and did not recur during one year (Figure 6). Whereas L30 was a superficial intramuscular lipoma in the thigh, ring chromosomes were identified in cytogenetic analysis. There was no recurrence in L30 during 3 years after surgery (Figure 7). In a histopathological review, L27 and L30 had a few hyperchromatic stromal cells within fibrous septa. Therefore, it is possible that L27 and L30 were actually cases of ALT/WDL. On the other hand, Nakayama et al. reported that *MDM2* amplification was frequently found in deep-seated intra- or inter-muscular lipomas [24].

Using total RNA samples, we could detect fusion genes by reverse transcription PCR as well as *MDM2* and *CDK4* expression levels by real-time PCR. This genetic profile is particularly useful for the differential diagnosis of ALT/WDL and lipoma.

In addition, while both *MDM2* and *CDK4* expression levels in core-needle biopsy sections were not significantly difference between lipoma and ALT/WDL (*MDM2*: P < 0.1, *CDK4*: P < 0.1, Mann–Whitney U test) (Figure 3), *MDM2* and *CDK4* expression levels in core-needle biopsy sections were compared to those in whole-tissue sections (*MDM2*: P = 0.6, *CDK4*: P = 0.8, Wiloxon signed-rank test) (MDM2: ρ = 0.827, P = 0.000001, CDK4: ρ = 0.746, P = 0.000001, Spearman rank correlation coefficient), which revealed no marked difference (Figure 4).

Because fast and useful methods that are applicable to core-needle biopsy are necessary in routine diagnosis, quantitative real-time PCR appears to be a reliable method for evaluating *MDM2* and *CDK4* gene expression in adipocytic tumors. Furthermore, using total RNA, and not DNA samples, the fusion genes of various sarcomas could be identified, such as *HMGA2-LPP* and *TLS-CHOP*, while detecting *MDM2* and *CDK4* overexpression by quantitative real-time PCR.

In the design of this study, there were two limitations of diagnosing adipocytic tumors by real-time PCR using total RNA. First, because of cytogenetic heterogeneity of adipocytic tumors, it is theoretically possible that real-time PCR using RNA may lead to both false-negatives and false-positives. Second, while the median levels of *MDM2* and *CDK4* expression were higher in ALT/WDL, the overlapping range of values for each tumor type is a limitation to the diagnostic usefulness of this test.

## Conclusions

The ease of use and reliability of real-time PCR when analyzing total RNA from core-needle biopsy sections makes it a potential routine diagnostic tool for liposarcoma. Furthermore, it may have potential use when diagnosing other cancers in which gene overexpression is a feature.

## Abbreviations

MDM2: Murine double-minute type 2; CDK4: Cyclin-dependent kinase 4; L: Lipoma; ALT/WDL: Atypical lipomatous tumors/well-differentiated liposarcomas; PCR: Polymerase chain reaction; DDL: Dedifferentiated liposarcomas; ISCN: International system for human cytogenetic nomenclature; MFH: Malignant fibrous histiocytoma; MPNST: Malignant peripheral nerve sheath tumor.

## Competing interests

The authors declare that they have no competing interests.

## Authors’ contributions

TS participated in the design of the study, conducted and evaluated the in vitro assay, performed the statistical analysis, and drafted the manuscript. AO contributed to the design of the study and helped to draft the manuscript. HK, TH, and HH participated in the design and coordination of the study. TA contributed to the design of the study and evaluated the in vitro assay. HU and RO conducted the pathological examination. NT contributed to the statistical analysis. TT conducted the cytogenetic analysis. NE participated in the design, evaluated the in vitro assay, and helped to draft the manuscript. All authors approved the final manuscript.

## Pre-publication history

The pre-publication history for this paper can be accessed here:

http://www.biomedcentral.com/1471-2407/14/468/prepub

## Supplementary Material

Additional file 1: Table S1 Summary of the performed methods. **Table S2.** Primer sequences of the fusion genes. **Table S3.** Primers used to amplify target genes. Click here for file
