# Supplementary material for: Real-time polymerase chain reaction analysis of MDM2 and CDK4 expression using total RNA from core-needle biopsies is useful for diagnosing adipocytic tumors
Source: BMC Cancer. 2014 Jun 26;14:468. doi: 10.1186/1471-2407-14-468 (PMC4075630; doi:10.1186/1471-2407-14-468)
Supplement: Additional file 1: Table S1 — Summary of the performed methods. Table S2. Primer sequences of the fusion genes. Table S3. Primers used to amplify target genes. [file 1471-2407-14-468-S1.doc]

**Supplemental materials**

**Additional file 1 Supplemental Table S1 -** Summary of the performed methods

| ***〈Total〉*** | ***Lipoma*** | ***ALT/WDL*** |
| --- | --- | --- |
| **168 cases** | **124/168** | **44/168** |
| **Cytogenetic findings** | | |
| ***104/168*** | ***66/104*** | ***38/104*** |
| ***HMGA2* fusion genes** | | |
| ***128/168*** | ***96/128*** | ***32/128*** |
| ***MDM2* and *CDK4* expression in whole tissue sections** | | |
| ***149/168*** | ***108/149*** | ***41/149*** |
| ***MDM2* and *CDK4* expression in core-needle biopsy sections** | | |
| ***38/168*** | ***28/38*** | ***10/38*** |

**Additional file 1 Supplemental Table S2 -** Primer sequences of the fusion genes

| **Primers** |  |
| --- | --- |
| **HMGIC-LPP fusion**  **HMG848F out**  **HMG878F in**  **LPP1980R in**  **LPP2064R out** | **5´-ACT TCA GCC CAG GGA CAA-3’**  **5´-GCG CCT CAG AAG AGA GGA C-3’**  **5´-GGG TAG CAG CCT TGG TTA TCT CCT-3’**  **5´-CTA AAG GTC AGT GCT CGC CTT G-3’** |
| **HMGIC-RDC1 fusion**  **HMG846F out**  **HMG876F in**  **HMGA2CHR2 in**  **HMGA2CHR2 out** | **5´-CCA CTT CAG CCC AGG GAC AAC C-3’**  **5´-CAG CGC CTC AGA AGA GAG GAC G-3’**  **5´-TGT GTC GAG CTG GTT CTG TAC GCG-3’**  **5´-TGG CAC TGA TAT TTT CTC GCC ATG-3’** |
| **HMGIC-NFIB fusion**  **HMG846F out**  **HMG876F in**  **NF1475-005R in**  **NF1475-006R out** | **5´-CCA CTT CAG CCC AGG CAC AAC C-3’**  **5´-CAG CGC CTC AGA AGA GAG GAC G-3’**  **5´-TGG CCG GTA AGA TGG GTC TCC T-3’**  **5´-TGG ACA TTG GCG GGT AAG ATG G-3’** |

**Additional file 1 Supplemental Table S3 -** Primers used to amplify target genes

| **Primers** |  |
| --- | --- |
| ***MDM2***  **forward primer**  **reverse primer** | **5´-TGG GCA GCT TGA AGC AGT TG-3’**  **5´-CAG GCT GCC ATG TGA CCT AAG A-3’** |
| ***CDK4***  **forward primer**  **reverse primer** | **5´-CTT CTG CAG TCC ACA TAT GCA ACA-3’**  **5´-CAA CTG GTC GGC TTC AGA GTT TC-3’** |
